# How to achieve quality assurance, shared ethics and efficient teambuilding? Lessons learned from interprofessional collaboration during the COVID-19 pandemic 

**DOI:** 10.3205/zma001372

**Published:** 2020-12-03

**Authors:** Jonathan Hunger, Heiko Schumann

**Affiliations:** 1 Maastricht University, Maastricht, The Netherlands; 2 Otto-von-Guericke- Universität Magdeburg, Medizinische Fakultät, Bereich Arbeitsmedizin, Magdeburg, Germany

**Keywords:** interprofessional cooperation, quality assurance, public health ethics, pandemic, training, mobile corona unit, COVID-19

## Abstract

**Objective:** Against the background of the current pandemic crisis, this case report presents the experiences made from interprofessional teamwork with group members from different medical qualification levels. Our objectives were

1. to identify areas of shared knowledge regarding efficient collaboration;
2. to improve effective teamwork based on mutual respect;
3. to develop innovative teaching methods tailored to the needs of COVID-19 interprofessional response teams.

**Methods: **Field notes from numerous team discussions and improvised internal training sessions were compiled into a checklist. Each author edited and revised the checklist and a consensus has been reached after an in-person discussion. Feedback from an academic expert in emergency services has been incorporated into the final version of the checklist.

**Results:** Three main topics were identified: the need for quality-assured professional training, the clarification of role expectations including assigned responsibilities, opportunities to contribute and participate in the team building process, and the development of area-related ethical competence in the sense of shared moral public health literacy. Hence, we developed the following ad - hoc teaching methods: use of online teaching videos, practical exercises on intubation models and the collective development of an annotated, detailed checklist for all relevant work processes of the mobile corona unit based on everyday debriefings.

**Summary:** The need for interprofessional team building in the context of the current health crisis provides a beneficial learning environment for all participants. We propose to conceptually refine this approach into a cross-professional, innovative method of teaching.

## Zusammenfassung

**Zielstellung:** Vor dem Hintergrund der aktuellen Pandemiekrise berichtet der vorliegende Projektbericht über Erfahrungen aus der interprofessionellen Teamarbeit mit Gruppenmitgliedern unterschiedlicher medizinischer Qualifikationsniveaus. Basierend auf den lessons learned werden relevante Wissensinhalte und Lehrformate vorgestellt, die sich für die effiziente und wertschätzende Zusammenarbeit als zielführend herausgestellt haben.

**Methoden:** Die Ergebnisse von regelmäßigen Teamdiskussionen und improvisierten internen Fortbildungen wurden in Form von field notes gesichert und zu einer Checkliste zusammengeführt, welche die Grundlage einer persönlichen Reflexion der Autoren und fachlichen Diskussion unter Einbezug einer akademisch – rettungsdienstlichen Perspektive bildete.

**Ergebnisse: **Es ließen sich drei inhaltliche Schwerpunkte herausarbeiten: die Notwendigkeit zur qualitätsgesicherten fachlichen Einarbeitung, die Klärung von Rollenerwartungen, Verantwortungsbereichen, Einfluss- und Entwicklungsmöglichkeiten im Teamprozess und die Entwicklung von arbeitsfeldbezogener Ethikkompetenz im Sinne einer *shared moral public health literacy*. Als ad hoc Lehrformate nutzen wir Online – Lernvideos, Übungen am Intubationsmodell, zielgruppenangepasste Fortbildungen und die gemeinsame Erarbeitung einer kommentierten, detaillierten Checkliste zu den Arbeitsabläufen der mobilen Corona-Einheit basierend auf täglichen Abschlussgesprächen.

**Zusammenfassung:** Die Notwendigkeit zur interprofessionellen Teambildung im Rahmen der aktuellen Gesundheitskrise stellt ein wertvolles Lernumfeld für alle Beteiligten dar. Wir schlagen vor, diesen Ansatz in einem professionsübergreifenden, innovativen Lehrformat aufzugreifen und konzeptionell weiterzuentwickeln.

## Introduction/background

As a response to numerous outbreak events in inpatient care facilities during the first months of the COVID-19 pandemic, mobile teams were progressively set up to perform on-site swab tests, initial medical assessments and basic advisory. These teams consisted of medical personnel, emergency workers and other (also non-medical) volunteers. They defined their operation as a medical service, meeting high standards of medical professionalism, especially in terms of quality assurance to avoid false negative results and likewise with regard to qualified counselling. In the context of infection control measures focusing on a population level, individual professional actions often had to be reflected against the medical ethical principles of “non – maleficence” and “patient´s autonomy” [1], especially when dealing with vulnerable patient groups.

Although medical care takes place in interdisciplinary processes and interprofessional contexts, the collaboration between various health care professions with different competencies is still poorly addressed in medical studies or in the training of emergency workers [2]. At the same time, the current pandemic crisis generates a considerable need for medical task forces in healthcare infrastructure, however often with discontinuous staffing. Lastly, due to the composition of the staff, these teams show a heterogeneous medical qualification level with different previous experiences in interprofessional teamwork. 

## Questions

- Which competencies and shared knowledge contribute to the efficiency of these interprofessional teams?
- Which training concepts can promote the competence development of the team members under the working conditions of the current critical situation?

## Methods

This project report from the founding phase and everyday work experience of a mobile corona unit is based on field notes (JH; student in the field of Public Health Management with professional experience as physicians) taken over a period of about three months (March-June 2020). We formulated lessons learned from the initial skill adaption training of new team members, frequent teaching sessions (duration approx. 10-15 min) and the results of continuous feedback rounds (individually between doctor and team member; daily in the operational subgroups). The unit had a total staff of 10 to 15 members with different medical qualification levels (level 2-8 according to the German Qualification Framework [3]), and was divided into 3-4 subgroups on a daily basis. Finally, a conclusive checklist summarizing the joint mission statement, working procedures and areas of responsibility was developed and refined in debriefing sessions with team leaders and the attendant medical doctor. Subsequently, prevailing topics were discussed with an academic expert (HS) with professional experience as paramedic and director of a Fire and Rescue College.

## Results

The following shared knowledge and competencies are particularly relevant for efficient and appreciative collaboration:

- knowledge of standard operating procedures: for example, how to do the swab test correctly or on the current level of information regarding hygiene measures and disease symptoms
- understanding of team processes: clarification of role expectations, areas of responsibility, active participation and involvement
- field-related ethical competence: ability to critically think in ethical conflict situations (e.g. indications for palliative patients, utilitarianisms and triage) in the sense of shared moral public health literacy [4].

However, it has been particularly challenging to assign roles and responsibilities to individual team members because of the fast-changing work environment, the different levels of professional expertise and inconsistent staffing. 

Team members’ organising skills acquired through previous experiences working for civilian relief agencies proved helpful, e.g., in developing on – site management structures and reporting channels. However, some staff members had to adjust to the flat organizational structures within the self-organised medical teams, but learned to appreciate them over time. We selected the following training formats:

- Quality-assured familiarization with swab testing on intubation training models and use of personal protective equipment, including explanatory videos from the online learning portal Amboss [https://www.amboss.com/de]. To comply with hygiene regulations, the video sequences were partly sent to the individual smartphones of the team members or laminated pocket cards were handed out.
- Short further education sessions with Q/A part and room for discussion, which served to integrate new information and latest scientific findings. The podcast Coronavirus-Update by the virologist Professor Christian Drosten was used as a valuable reference [https://www.ndr.de/nachrichten/info/podcast4684.html].
- Compilation of an annotated, detailed checklist on the workflows and shared guiding principles for the mobile corona units.

**Figure 1:**
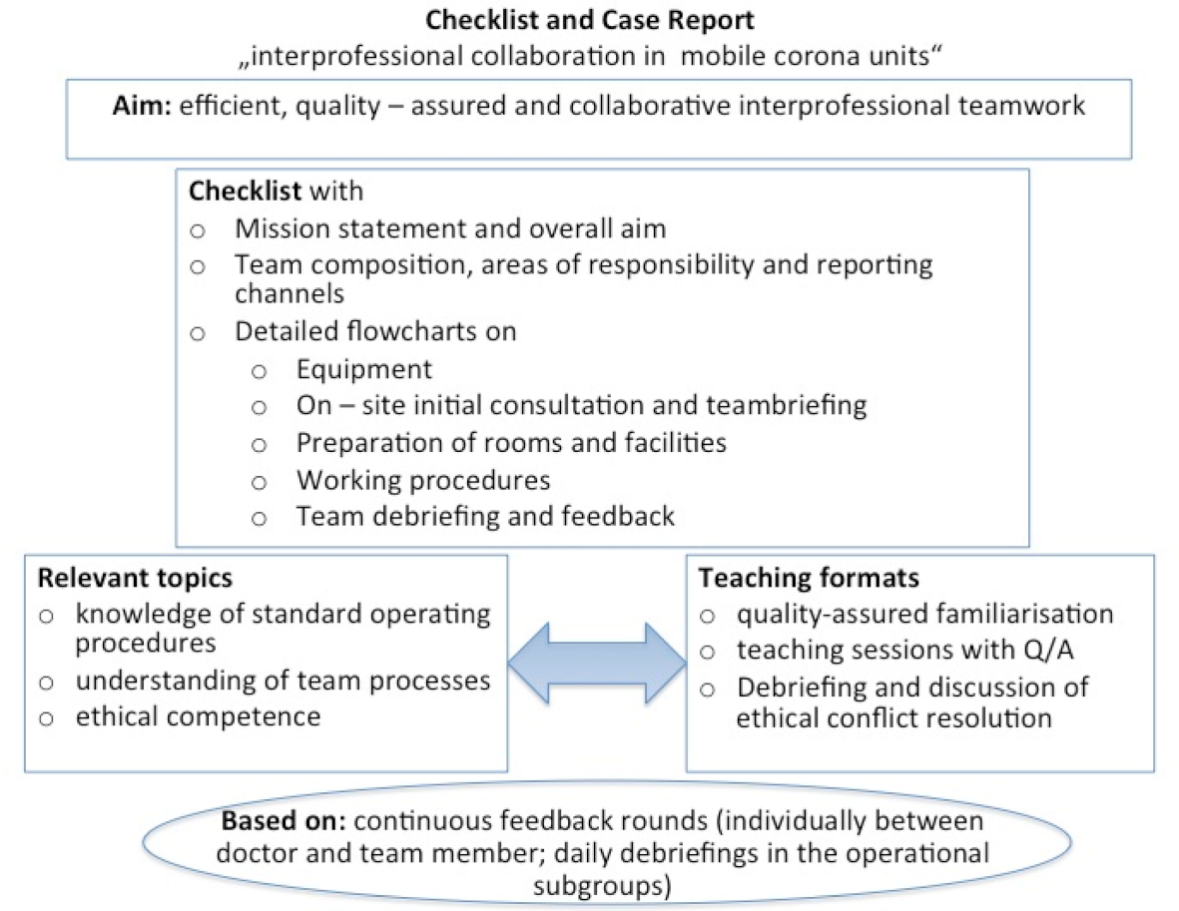
Synopsis of concepts and methods contributing to efficient interprofessional teamwork in mobile corona units (author's visualisation)

The outlined topics, training methods and contents of the checklist are summarized in the following illustration (see figure 1 (Fig. 1)).

## Discussion

To our knowledge, this is the first conclusive presentation of training elements for improving inter-professional cooperation in mobile corona units, based on practical experience and created under COVID-19 pandemic conditions. Given the prevailing shortage of time and inconsistency in staffing, our checklist contributed to the improvement of on – site workflow, training, organisational and time efficiency skills. Our mission statement based on shared values and ethical principles strengthened the collective identity and increased participation and motivation.

## Conclusion

In the context of the current health crisis, the presented topics and training elements for imparting interprofessional knowledge contribute to an efficient and appreciative interprofessional collaboration. With regards to future challenges, we propose a conceptual refinement and subsequent evaluation of our first lessons learned.

## Competing interests

The authors declare that they have no competing interests.
